# Incidental Lipid Poor Adrenal Mass in a Patient with Antiphospholipid Syndrome

**DOI:** 10.1155/2013/379852

**Published:** 2013-02-20

**Authors:** Subramanian Kannan, Ankita Satra, Amir Hamrahian

**Affiliations:** ^1^Department of Endocrinology, Diabetes and Metabolism, Cleveland Clinic Foundation, 9500 Euclid Avenue Desk F20, Cleveland, OH 44195, USA; ^2^Department of Internal Medicine, Cleveland Clinic Foundation, 9500 Euclid Avenue Desk F20, Cleveland, OH 44195, USA

## Abstract

Adrenal incidentalomas are commonly encountered in this era of ubiquitous imaging. The attenuation of the incidentaloma measured in Hounsfield units (HU) is an important step in the work up. Attenuation less than 10 HU indicates a benign lesion in more than 98% of cases, whereas attenuation greater than 30 HU is highly suspicious for adrenocortical cancer (ACC). Adrenal hematoma is rarely suspected clinically and exhibits no specific clinical symptoms or laboratory findings. There are multiple radiological features of adrenal hemorrhage and can mimic ACC. We present a case of an adrenal mass in a patient with antiphospholipid syndrome and discuss radiological clues to differentiate adrenal hematomas from ACC and thus avoid unnecessary surgical intervention.

## 1. Case Report

**Figure 1:**
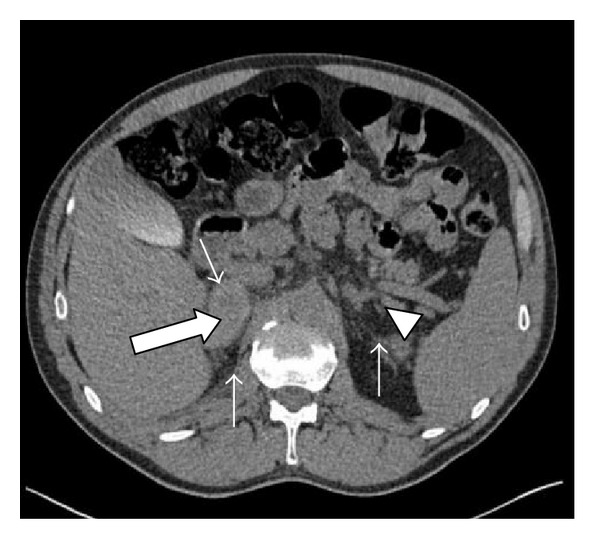
Adrenal CT without contrast reveals right adrenal mass (thick arrow) and thickening of the left adrenal gland (arrow head). Soft tissue stranding is indicated by the thin arrows.

**Figure 2:**
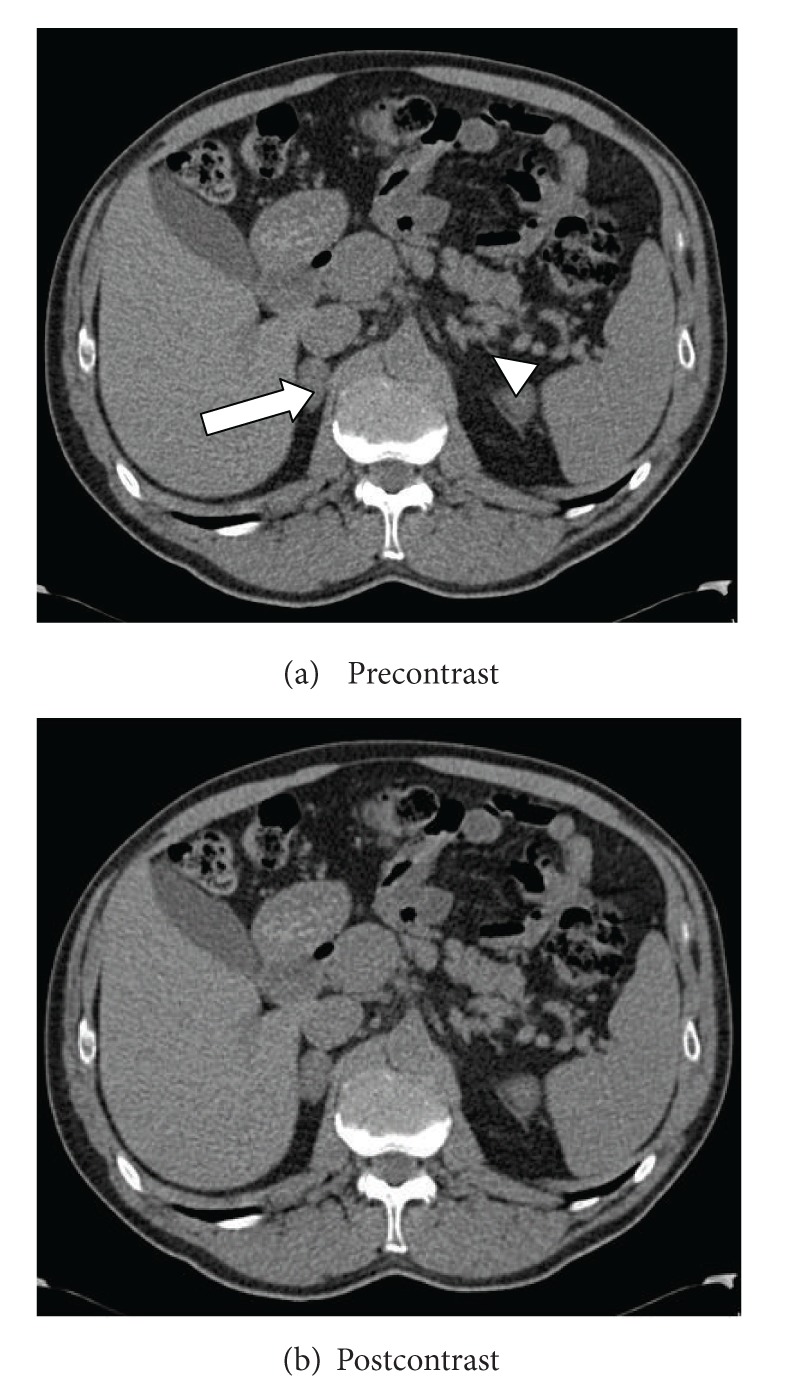
(a) Adrenal CT without contrast demonstrating a reduction in the size and the attenuation value of right adrenal mass (thick arrow); normal left adrenal gland (arrow head); (b) postcontrast image reveal no enhancement.

A 65-year-old male with past history of anti-phospholipid (APL) syndrome on coumadin was admitted for acute right sided pleuritic chest pain. He was recently bridged with low molecular weight heparin after a right knee replacement. The CT-angiogram of the chest revealed a pulmonary infarct and a 5 cm right adrenal mass. A dedicated CT of his adrenals confirmed a right adrenal mass of 5.2 × 3.1 cm with pre-contrast attenuation of 53 Hounsfield units (HU) (Figure 1) with evidence of fat stranding, a sign of blood infiltrating fat containing retroperitoneal soft tissue, surrounding it. The left adrenal gland was thickened. A review of the patient's imaging studies was notable for normal adrenal glands on a CT scan two years ago. Lab work-up revealed a cortisol level of 4.4 mcg/dL after 1 mg dexamethasone suppression test, a normal plasma ACTH 28 pg/mL (8–42), and a slightly elevated plasma normetanephrine 1.2 nmol/L (<0.9). Given the clinical circumstances of APL syndrome, history of anticoagulation and radiological features, a close monitoring of the adrenal mass was planned. A repeat CT scan at 2 months showed a decrease in right adrenal mass and noncontrast CT attenuation value to 4.5 × 2.6 cm and 32 HU, respectively. Another CT scan done at 4 months showed 50% reduction in size (2.6 × 1.4 cm) and attenuation reduced to 21 HU (Figure 2(a)). The CT washout showed no significant enhancement or de-enhancement (Figure 2(b)). Left adrenal gland appeared normal. What is the diagnosis?

## 2. Discussion

The various causes of an incidentally detected adrenal mass are listed in Table 1. Patient's adrenal mass imaging characteristics are consistent with an adrenal hematoma. The 1 mg dexamethasone suppression test was repeated on outpatient basis and subclinical cushing's syndrome was ruled out. 

Adrenal hematomas may present as a solid mass with high attenuation value (50–90 HU) on CT scan. In contrast to adrenal metastasis and adrenocortical carcinoma they do not enhance with contrast and therefore have a very low washout percentage [1]. Adrenal hematomas decrease in size and attenuation on follow-up imaging and the majority undergo complete resolution [2]. The appearance of an adrenal hematoma on MRI evolves predictably over time due to the different components of acute, subacute, and chronic hemorrhage (deoxyhemoglobin, methemoglobin, and hemosiderin).

Acute adrenal hematomas appear isointense to hypointense relative to liver on T1-weighted images and hypointense on T2-weighted images due to deoxyhemoglobin. Subacute hematomas appear heterogeneous and hyperintense on T1- and T2-weighted images due to methemoglobin, while chronic hematomas exhibit peripheral hypointensity due to the susceptibility effects from the accumulation of hemosiderin and development of a fibrous capsule [4]. Other patterns of adrenal hematoma include infiltrative pattern where there is soft tissue stranding in the retroperitoneum, adreniform pattern with enlargement of the limbs of adrenal gland, and a solid mass with cystic center. The latter may also suggest the presence of hemorrhage in an underlying mass and prompts surgical excision for conclusive diagnosis [2]. Bilateral adrenal hemorrhages may present with primary adrenal insufficiency [5]. Consideration of adrenal hematoma in the differential diagnosis of a lipid poor adrenal mass is very important since unnecessary surgical intervention can be avoided. 

Follow-up imaging in 2-3 months is necessary to confirm the diagnosis and rules out malignant adrenal masses. 

## Figures and Tables

**Table 1:** Various causes of adrenal incidentalomas [3].

(a) Adrenal cortical tumors	
Adenoma, nodular hyperplasia, and congenital adrenal hyperplasia	
Carcinoma	
(b) Adrenal medullary tumors	
Pheochromocytoma	
Ganglioneuroma/neuroblastoma	
(c) Other adrenal tumors	
Myelolipoma	
Metastases	
Miscellaneous, for example, hamartoma, teratoma, lipoma, hemangioma	
Infections:	
Fungal: (histoplasmosis, coccidiomycosis, blastomycosis)	
Viral: CMV	
Parasitic	
Granulomas	
Tuberculosis	
Sarcoidosis	
Cysts and pseudocysts	
Hematomas	
